# Heat-shock proteins and mRNAs in liver and hepatoma.

**DOI:** 10.1038/bjc.1987.130

**Published:** 1987-06

**Authors:** L. Bardella, L. Schiaffonati, G. Cairo, A. Bernelli-Zazzera

## Abstract

**Images:**


*Br. J. Cancer* (1987), **55**, 643–645 © The Macmillan Press Ltd., 1987

SHORT COMMUNICATION

### Heat-shock proteins and mRNAs in liver and hepatoma

L. Bardella, L. Schiaffonati, G. Cairo & A. Bernelli-Zazzera

*Istituto di Patologia Generale dell'Università degli Studi di Milano, Centro di Studio sulla Patologia Cellulare del CNR, Via Mangiagalli 31, 20133 Milano, Italy.*

Eukaryotic cells, exposed to high temperature, undergo a complex series of changes of their metabolic activities, which are comprehensively known as heat shock response. The main feature of this response is the rapid shift to the preferential synthesis of a set of proteins, the heat shock proteins (hsp), in a context of depressed overall protein synthesis. Hsp, and in particular the most common and abundant among them (hsp 70), are highly conserved in the different species (Schlesinger *et al.*, 1982). The accumulation of hsp has been related to the development of thermotolerance, which seems to be induced even when hsp synthesis is triggered by stressful conditions other than heat (Li, 1983); but new findings on the appearance of hsp in connection with viral transformation (Nevins, 1982; Imperiale *et al.*, 1984), development (Morange *et al.*, 1984) and cell cycle (Kao *et al.*, 1985) suggest that they might have a more fundamental role in the life of the cells, in particular in cell growth and differentiation. Transcription of the mammalian hsp 70 gene is elevated in several tumour cell lines, and hsp 70 synthesis is induced by serum stimulation in human cells in culture (Imperiale *et al.*, 1984; Kao *et al.*, 1985; Wu & Morimoto, 1985). The purpose of the present study is to examine hsp synthesis in liver and in a transplantable hepatoma in order to detect possible alterations in hsp synthesis in cancer cells: the simultaneous analysis of the population of mRNAs in the tumours can add further information as to the mechanisms of control of the synthesis of hsp in normal and neoplastic tissues.

Normal and tumour-bearing rats were made hyperthermic *in vivo* by amphetamine treatment and environmental high temperature, as previously described (Cairo *et al.*, 1985). The fast-growing Morris hepatomas 3924A (time betwen transplantations: 22–25 days) were used at half the average transfer time of the tumours. Liver and hepatoma slices from normothermic and hyperthermic rats were labelled for 1 h at 37°C in the presence of $^{35}$S-methionine. Incubation conditions and preparations of samples for analysis by 1-D gel electrophoresis were as described (Bardella *et al.*, 1986). Total liver and hepatoma RNAs were isolated as described by Raymond and Shore (1979). Cell-free protein synthesis was performed in a rabbit reticulocyte lysate in the presence of $^{35}$S-methionine using a commercially available kit (Amersham). *In vitro* labelled proteins were subjected to 2-D gel electrophoresis. 1-D and 2-D gel electrophoresis were performed as described (Cairo *et al.*, 1985). Protein samples of equal radioactivity were loaded on the gels that were processed for fluorography (Laskey & Mills, 1975). The relative fraction of each band was obtained from the densitometric tracing of the fluorograms of 1-D gel electrophoresis as described (Cairo *et al.*, 1985). Total cellular RNA was electrophoresed through a 1.5% agarose, 6% formaldehyde gel, blotted to nitrocellullose filter and probed with the coding region of Drosophila hsp 70 gene from plasmid pPW229, in 50% formamide, 450 mM NaCl, 45 mM Na citrate, 0.1% bovine serum albumin, 0.1% Ficoll, 0.1% polyvinylpyrrolidone, 0.1% SDS, 50 mM phosphate, pH 6.5, 0.1 mg ml$^{-1}$ ssDNA at 37°C for 24 h. The amount of RNA in each lane was corrected by the amount of rRNA as determined by hybridization of pXCR7 probe to the same filter. The cDNAs were labelled by nick translation with $^{32}$P dCTP using a commercially available kit (Amersham).

The electrophoretic patterns of hepatoma (Figure 1) obviously differ from the liver; the analysis of these changes, which were expected and reflect differences in gene expression in the tumour, but which do not concern hsp, is beyond the purpose of the present investigation. Heat-shock promotes the synthesis of some new proteins without marked simplification of the previous pattern. The same proteins are induced in slices of both liver and hepatoma from normothermic rats heated *in vitro* (Bardella *et al.*, 1986) thus indicating that the treatment applied to the rats actually increases the temperature of the tissues. Comparison with the standards indicates that these new proteins correspond to the hsp group of 89 and 70 molecular weight. Quantitative values of the relative rate of synthesis of the induced and constitutive hsp, are also shown in Figure 1. The synthesis of hsp 70 and 89 is strongly induced in liver slices from hyperthermic animals. In the 70 kd area we could not find any constitutive or slicing-induced hsp, which was reported in significant amount in several mammalian tissues, brain in particular (Currie & White, 1983). The band of 69 kd found in these gels is presumably albumin. Although 89 hsp synthesis has been reported in many mammalian tissues in the absence of stress (Schlesinger, 1986), we could not detect any 89 hsp under basal conditions. On the other hand, the pattern of labelling of our liver slices is superimposable to the one obtained by labelling experiments *in vivo* (Bardella *et al.*, 1984), and corresponds to the pattern of proteins synthesized *in vitro* by rat liver poly (A$^+$) RNA (Cairo *et al.*, 1985). As a difference from normal liver, 3924A Morris hepatoma synthesizes hsp constitutively, i.e. also before heat-shock: the basal level of hsp 89 synthesis is further enhanced by heat-shock, but less than in liver. The hsp 70 is also expressed before, and only slightly induced by heat-shock. The band at the migration position of albumin is less pronounced in hepatoma, in keeping with the reduced albumin synthesis in liver tumours (Schreiber *et al.*, 1966). In agreement with this fact, hybridization experiments (not shown) revealed a very low amount of albumin mRNA in this hepatoma. The induced formation of hsp depends usually on the synthesis of new mRNA species: we used an *in vitro* translation system to probe hsp mRNAs levels by means of 2-D electrophoretic analysis of their translational products. In the liver of hyperthermic rats (Figure 2) both hsp 89 and 70 mRNAs are induced, and the products of the latter appear in several isoforms. As a difference from liver, 3924A hepatoma synthesizes hsp mRNAs constitutively, and the effect of heat-shock on their synthesis is less pronounced; in addition, only the more acidic forms of hsp 70 are synthesized by hepatoma mRNA under both circumstances. The correlation between the level of hsp 89 and 70 and the amount of their translatable mRNAs suggests a transcriptional regulation of hsp synthesis both in liver and in hepatoma. In a further group of experiments we made Northern blot analysis of total cellular RNA from both liver

Correspondence: A. Bernelli-Zazzera.
Received 25 November 1986; and in revised form, 9 February 1987.

**Figure 1** Heat shock protein synthesis by tissue slices from liver and 3924A Morris hepatoma. Fluorograms of 1-D gel electrophoresis are shown on the left of the figure. Equal amounts of peptides labelled *in vitro* were applied to each of the gel slots. Bars on the right of the gel represent the migration position of $^{14}C$ methylated marker proteins run in a parallel slot of the gel: 97,000, phosphorylase B; 69,000, bovine serum albumin; 46,000, ovalbumin, from the top to the bottom. Arrows on the left of the gel indicate the position of heat shock proteins. c: slices from normothermic rat; hs: slices from hyperthermic rat. Histograms of the relative amount of heat shock proteins (% of total proteins) are shown on the right of the figure. The values were obtained from densitometric tracings of the gels.

**Figure 2** Fluorograms of 2-D gel electrophoresis of the translational products of total RNA from liver and 3924A Morris hepatoma. (a) liver from normothermic rat. (b) liver from hyperthermic rat. (c) hepatoma from normothermic rat. (d) hepatoma from hyperthermic rat. Upward arrows ↑ indicate the migration position of HSP 89. Downward arrows ↓ indicate the migration position of HSP 70 family.

and hepatoma with a hsp 70 Drosophila probe (Figure 3). Heterologous hybridization is feasible because of the high similarity of hsp 70 from different organisms also at the DNA level (Hunt & Morimoto, 1985). Normal rat liver expresses, at a barely detectable level, only one species of RNA homologous to the Drosophila hsp 70 probe: upon heat-shock the expression of this mRNA is considerably enhanced and two new slower-migrating bands appear. In the 3924A Morris hepatoma the fast-migrating mRNA species is present in high amount also before heat-shock and is induced less than in liver: also the two slow migrating mRNAs increase much less than in normal liver following heat-shock. Three bands homologous to the Drosophila hsp 70 gene have been detected also in heat-shocked mouse L cells (Lowe & Moran, 1984). These observations are in agreement with the fact that hsp 70 gene belongs to a family coding for similar but differently regulated products: some members of the family are heat inducible while others, the so called 'heat-shock cognate genes', are transcribed at normal temperature (Ingolia & Craig, 1982). The different patterns of protein translated *in vitro* and of RNA species hybridizing to the Drosophila probe seem to suggest that the hsp 70 gene is differently regulated in liver and in the hepatoma. The slow migrating mRNAs, induced at different extent in liver and in tumours could code for the heat inducible hsp 70 and/or for the GRP 78 (Munro & Pelham, 1986). The fast migrating band, which is less responsive to induction and is present also before heat-shock in both tissues but at higher level in the hepatoma, could represent an RNA species related more to cell growth than to heat shock response proper and possibly coding for the more acidic hsp 70 isoforms. The high constitutive hsp synthesis in the hepatoma

**Figure 3** Northern blot analysis of RNA from liver and 3924A Morris hepatoma. 25 μg of total RNA were loaded and probed with a 2.6 kd XhoI DNA fragment containing the amino acid coding region of hsp 70 Drosophila gene. Control: RNA extracted from tissues of normothermic rats. Heat shock: RNA extracted from tissues of hyperthermic rats.

might be related to the high growth rate possibly supported by some oncogene products (Kingston *et al.*, 1984; Kao *et al.*, 1985), or to particular conditions associated with protein assembly or degradation (Pelham, 1986). The defective induced synthesis of hsps in hepatoma cells can be interpreted on the basis of observations that the heat-shock response is self-regulated at both the transcriptional and post-transcriptional levels (Di Domenico *et al.*, 1982); high pre-existing amounts of hsps could inhibit the response caused by the heat-shock inducing treatment.

We thank Professor T. Galeotti, Università Cattolica del S. Cuore, Roma, for supplying the transplantable hepatomas; Dr. U. Cornelli of Recordati S.p.A. for a gift of d-amphetamine sulphate; Mr V. Albini for technical assistance and Ms M.G. Bombonato for typing the manuscript. The pXCR7 clone for rDNA of *Xenopus laevis* and the pPW229 clone for Drosophila hsp 70 gene were generous gifts of Dr I. Bozzoni and Dr M. Meselson respectively. Work supported by grant 84.00447.044 of CNR P.F. Oncologia, presented in part at the 618th Meeting of the Biochemical Society.

### References

BARDELLA, L., CAIRO, G., SCHIAFFONATI, L. & BERNELLI-ZAZZERA, A. (1984). Post-ischemic recovery and acute phase reaction in the liver cells. In *Frontiers in Gastrointestinal Research*, **8**, p. 63. Karger: Basel.

BARDELLA, L., CAJONE, F., CAIRO, G., SCHIAFFONATI, L. & BERNELLI-ZAZZERA, A. (1986). Synthesis of heat shock proteins and tumor growth. *Toxicologic Pathology*, **14**, 353.

√ CAIRO, G., BARDELLA, L., SCHIAFFONATI, L. & BERNELLI-ZAZZERA, A. (1985). Synthesis of heat shock proteins in rat liver after ischemia and hyperthermia. *Hepatology*, **5**, 357.

CURRIE, R.W. & WHITE, F.P. (1983). Characterization of the synthesis and accumulation of a 71 kilodalton protein induced in rat tissues after hyperthermia. *Can. J. Biochem. Cell. Biol.*, **61**, 438.

DI DOMENICO, B.J., BUGAISKY, G.E. & LINDQUIST, S. (1982). The heat shock response is self regulated at both the transcriptional and post-transcriptional levels. *Cell*, **31**, 593.

HUNT, C. & MORIMOTO, R.I. (1985). Conserved features of eukaryotic hsp 70 genes revealed by comparison with the nucleotide sequence of human hsp 70. *Proc. Natl Acad. Sci. USA*, **82**, 6455.

IMPERIALE, M.J., KAO, H.T., FELDMAN, L.T., NEVINS, J.R. & STRICKLAND, S. (1984). Common control of the heat shock gene and early adenovirus genes: evidence for a cellular E1A-like activity. *Mol. Cell. Biol.*, **4**, 867.

INGOLIA, T.D. & CRAIG, E.A. (1982). Drosophila gene related to the major heat shock induced gene is transcribed at normal temperatures and not induced by heat shock. *Proc. Natl Acad. Sci. USA*, **79**, 525.

KAO, H.T., CAPASSO, O., HEINTZ, N. & NEVINS, J.R. (1955). Cell cycle control of the human HSP 70 gene: implications for the role of a cellular E1A-like function. *Mol. Cell. Biol.*, **5**, 628.

KINGSTON, R.F., BALDWIN, A.S. JR. & SHARP, P.A. (1984). Regulation of heat shock gene 70 expression by c-myc. *Nature*, **312**, 280.

LASKEY, R.A. & MILLS, A.D. (1975). Quantitative film detection of $^{3}H$ and $^{14}C$ in polyacrylamide gels by fluorography. *Eur. J. Biochem.*, **56**, 335.

LI, G.C. (1983). Induction of thermotolerance and enhanced heat shock protein synthesis in Chinese hamster fibroblasts by sodium arsenite and by ethanol. *J. Cell. Physiol.*, **115**, 116.

LOWE, D.G. & MORAN, L.A. (1984). Proteins related to the mouse L-cell major heat shock protein are synthesized in the absence of heat shock gene expression. *Proc. Natl Acad. Sci. USA*, **81**, 2317.

√ MORANGE, M., DIU, A., BENSAUDE, O. & BABINET, C. (1984). Altered expression of heat shock proteins in embryonal carcinoma and mouse early embryonic cells. *Mol. Cell. Biol.*, **4**, 730.

MUNRO, S. & PELHAM, H.R.B. (1986). An Hsp 70-like gene protein in the ER: identity with the 78 kd glucose-regulated protein and immunoglobulin heavy chain binding protein. *Cell*, **46**, 291.

NEVINS, J.R. (1982). Induction of the synthesis of 70.000 dalton mammalian heat shock protein by the adenovirus E1A gene product. *Cell*, **29**, 913.

PELHAM, H.R.B. (1986). Speculations on the functions of the major heat shock and glucose-regulated proteins. *Cell*, **46**, 959.

RAYMOND, Y. & SHORE, G.C. (1979). The precursor for carbamyl phosphate synthetase is transported to mitochondria via a cytosolic route. *J. Biol. Chem.*, **19**, 9335.

SCHLESINGER, M.J., ASHBURNER, M. & TISSIERES, A. (1982). *Heat Shock From Bacteria to Man*. Cold Spring Harbor Laboratory: Cold Spring Harbor, NY.

SCHLESINGER, M.J. (1986). Heat shock proteins: the search for functions. *J. Cell Biol.*, **103**, 321.

SCHREIBER, G., BOUTWELL, R.K., POTTER, V.R. & MORRIS, H.P. (1966). Lack of secretion of serum protein by transplanted rat hepatomas. *Cancer Res.*, **26**, 2357.

WU, B.J. & MORIMOTO, R.I. (1985). Transcription of the human hsp 70 gene is induced by serum stimulation. *Proc. Natl Acad. Sci. USA*, **82**, 6070.


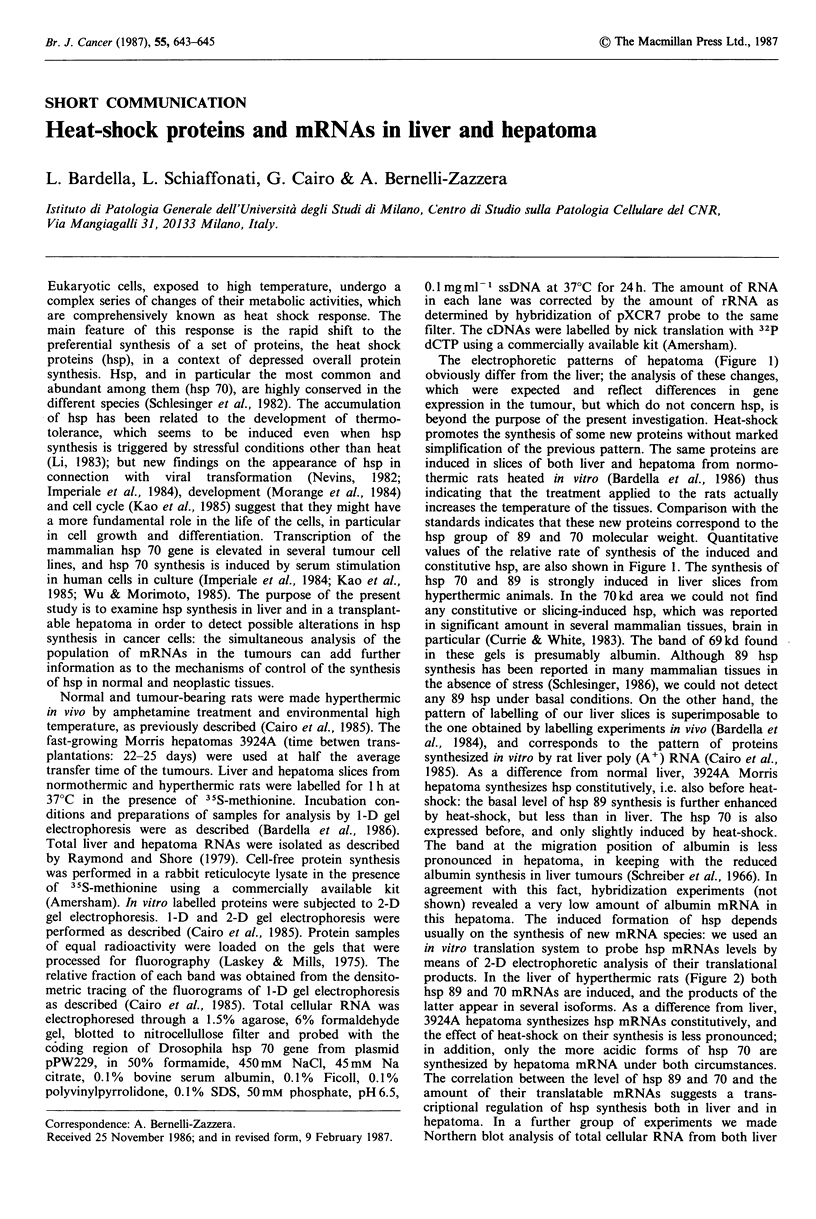

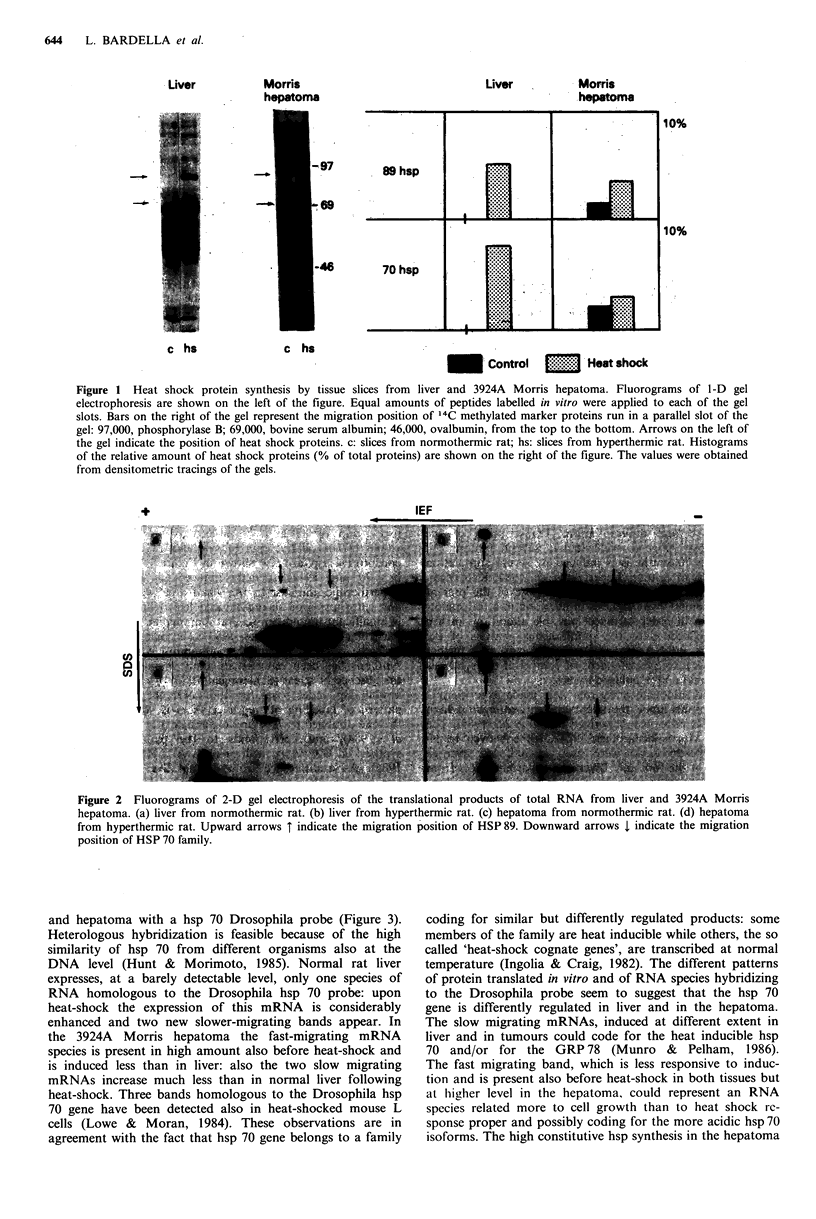

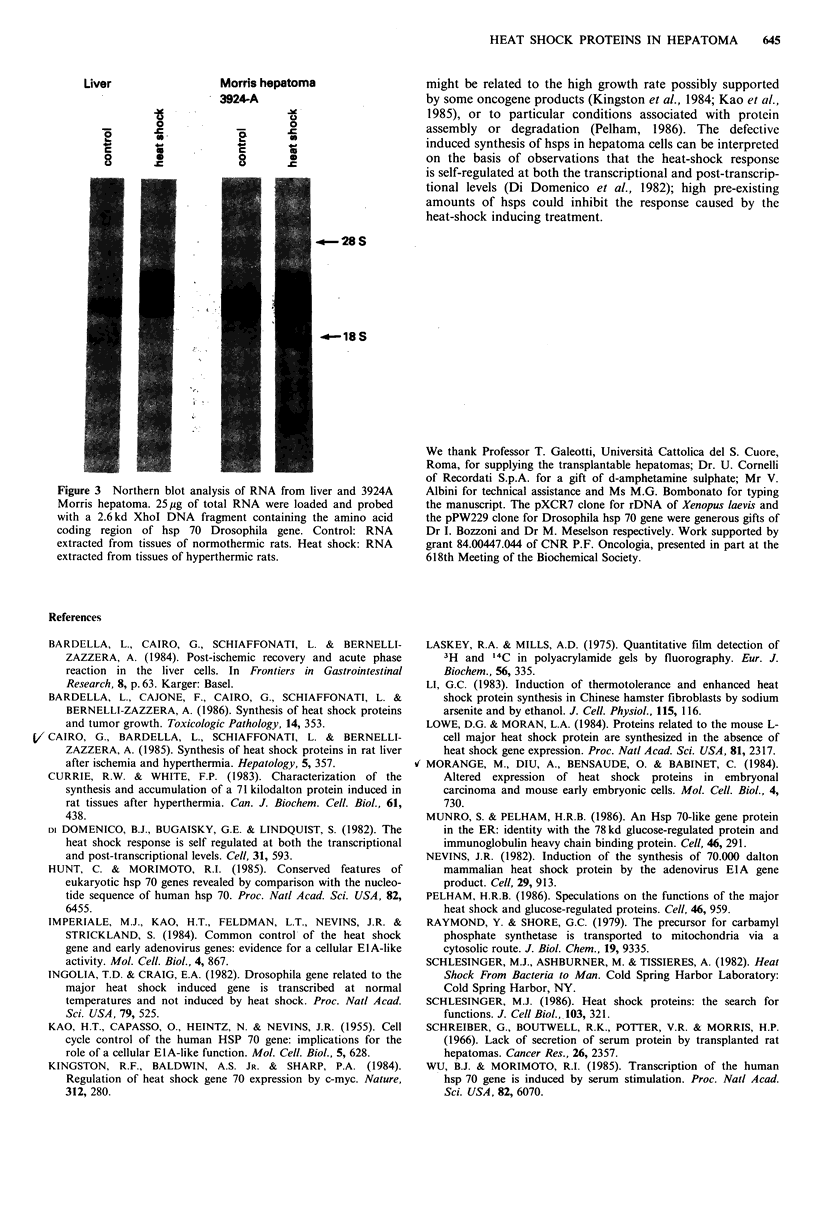


## References

[OCR_00265] Bardella L., Cajone F., Cairo G., Schiaffonati L., Bernelli-Zazzera A. (1986). Synthesis of heat-shock proteins and tumor growth.. Toxicol Pathol.

[OCR_00272] Cairo G., Bardella L., Schiaffonati L., Bernelli-Zazzera A. (1985). Synthesis of heat shock proteins in rat liver after ischemia and hyperthermia.. Hepatology.

[OCR_00275] Currie R. W., White F. P. (1983). Characterization of the synthesis and accumulation of a 71-kilodalton protein induced in rat tissues after hyperthermia.. Can J Biochem Cell Biol.

[OCR_00281] DiDomenico B. J., Bugaisky G. E., Lindquist S. (1982). The heat shock response is self-regulated at both the transcriptional and posttranscriptional levels.. Cell.

[OCR_00286] Hunt C., Morimoto R. I. (1985). Conserved features of eukaryotic hsp70 genes revealed by comparison with the nucleotide sequence of human hsp70.. Proc Natl Acad Sci U S A.

[OCR_00292] Imperiale M. J., Kao H. T., Feldman L. T., Nevins J. R., Strickland S. (1984). Common control of the heat shock gene and early adenovirus genes: evidence for a cellular E1A-like activity.. Mol Cell Biol.

[OCR_00298] Ingolia T. D., Craig E. A. (1982). Drosophila gene related to the major heat shock-induced gene is transcribed at normal temperatures and not induced by heat shock.. Proc Natl Acad Sci U S A.

[OCR_00304] Kao H. T., Capasso O., Heintz N., Nevins J. R. (1985). Cell cycle control of the human HSP70 gene: implications for the role of a cellular E1A-like function.. Mol Cell Biol.

[OCR_00309] Kingston R. E., Baldwin A. S., Sharp P. A. (1984). Regulation of heat shock protein 70 gene expression by c-myc.. Nature.

[OCR_00314] Laskey R. A., Mills A. D. (1975). Quantitative film detection of 3H and 14C in polyacrylamide gels by fluorography.. Eur J Biochem.

[OCR_00319] Li G. C. (1983). Induction of thermotolerance and enhanced heat shock protein synthesis in Chinese hamster fibroblasts by sodium arsenite and by ethanol.. J Cell Physiol.

[OCR_00324] Lowe D. G., Moran L. A. (1984). Proteins related to the mouse L-cell major heat shock protein are synthesized in the absence of heat shock gene expression.. Proc Natl Acad Sci U S A.

[OCR_00328] Morange M., Diu A., Bensaude O., Babinet C. (1984). Altered expression of heat shock proteins in embryonal carcinoma and mouse early embryonic cells.. Mol Cell Biol.

[OCR_00334] Munro S., Pelham H. R. (1986). An Hsp70-like protein in the ER: identity with the 78 kd glucose-regulated protein and immunoglobulin heavy chain binding protein.. Cell.

[OCR_00339] Nevins J. R. (1982). Induction of the synthesis of a 70,000 dalton mammalian heat shock protein by the adenovirus E1A gene product.. Cell.

[OCR_00344] Pelham H. R. (1986). Speculations on the functions of the major heat shock and glucose-regulated proteins.. Cell.

[OCR_00348] Raymond Y., Shore G. C. (1979). The precursor for carbamyl phosphate synthetase is transported to mitochondria via a cytosolic route.. J Biol Chem.

[OCR_00358] Schlesinger M. J. (1986). Heat shock proteins: the search for functions.. J Cell Biol.

[OCR_00362] Schreiber G., Boutwell R. K., Potter V. R., Morris H. P. (1966). Lack of secretion of serum protein by transplanted rat hepatomas.. Cancer Res.

[OCR_00367] Wu B. J., Morimoto R. I. (1985). Transcription of the human hsp70 gene is induced by serum stimulation.. Proc Natl Acad Sci U S A.

